# Molecular analyses of prostate tumors for diagnosis of malignancy on fine-needle aspiration biopsies

**DOI:** 10.18632/oncotarget.22289

**Published:** 2017-11-06

**Authors:** Menglin Shan, Qianlin Xia, Dong Yan, Yanjun Zhu, Xuan Zhang, Guihong Zhang, Jianming Guo, Jun Hou, Weiping Chen, Tongyu Zhu, Xiaoyan Zhang, Jianqing Xu, Jin Wang, Tao Ding, Jianghua Zheng

**Affiliations:** ^1^ Shanghai Public Health Clinical Center, Fudan University, Jinshan, Shanghai, P.R. China; ^2^ Department of Medical Oncology, Beijing Chaoyang Hospital Affiliated to Capital Medical University, Beijing, P.R. China; ^3^ Department of Urology, Zhongshan Hospital, Fudan University, Yangpu, Shanghai, P.R. China; ^4^ Pathology, Zhongshan Hospital, Fudan University, Yangpu, Shanghai, P.R. China; ^5^ Genomics Core, National Institute of Diabetes and Digestive and Kidney Diseases, National Institutes of Health, Bethesda, MD, USA; ^6^ Department of Urology, The Sixth People's Hospital South Campus, Shanghai Jiao Tong University, Fengxian, Shanghai, P.R. China; ^7^ Department of Laboratory Medicine, Zhoupu Hospital Affiliated to Shanghai University of Medicine and Health Sciences, Pudong New Area, Shanghai, P.R. China

**Keywords:** prostate cancer, fine-needle aspiration, gene expression profiling, pathway analysis

## Abstract

Prostate cancer (PCa) is a common cancer and remains the second-leading cause of cancer-associated mortality in men, but diagnosis of PCa remains a main clinical challenge. To investigate the involvement of differentially expressing genes in PCa with deregulated pathways to allow earlier diagnosis of the disease, transcriptomic analyses of differential expression genes in fine-needle aspiration (FNA) biopsies helped to discriminate PCa from benign prostatic hyperplasia (BPH). We identified 255 genes that were deregulated in prostate tumors compared with BPH tissues. qRT-PCR was conducted to examine the expression levels of the four genes in FNA biopsies and confirmed that ITGBL1 was significantly up-regulated and HOXA7, KRT15 and TGM4 were down-regulated in the PCa compared to the BPH, with a sensitivity of 87.1% and a specificity of 87.8%; the area under the receiver operating characteristic curve was estimated at 0.94, which was significantly improved compared with PSA alone (AUC = 0.82). Moreover, the increased expression of ITGBL1 correlated with total cholesterol, triglyceride and PSA. Our results demonstrated that transcriptomic analyses in FNA biopsies could facilitate rapid identification of potential targets for therapy and diagnosis of PCa.

## INTRODUCTION

Prostate cancer (PCa) is the second most common cause of death from malignancies among men in the world. There will be approximately 161,360 new cases diagnosed and 26,730 deaths due to PCa in 2017, which represent 19% of all cancer cases and 8% of cancer-related deaths among men in the USA, respectively [1]. Furthermore, the incidence rates of PCa in China also have been increasing dramatically recently; primary causes of death for PCa patients are invasion and metastasis [2, 3]. Until now, the decisive diagnosis of PCa mainly depending on prostate biopsy analysis [4], which can provide false negative results especially when the tumor size is small in the early PCa stage. Focal heterogeneity and multifocal presentation of PCa also may lead to sampling errors for the prognostic assessment. Current common screening techniques are based on the quantification of serum prostate specific antigen (PSA) levels and the digital rectal examination [5]. PSA, known as kallikrein-3 (KLK3), is a marker for prostate cells [6], but not specific for PCa. Currently, PSA is used for early detection of PCa and follow-up during hormone therapy or after surgery. Moreover, during chemical castration, an increasing PSA level can indicate therapy failure. Previous studies have shown that serum PSA levels above 4.0 ng/ml indicated a possible sign of PCa, and PSA levels of 4.0 ng/ml and lower were considered normal [7]. PSA velocity, the increase over time of PSA circulating levels, has been proposed as a more specific marker for PCa [8]. However, approximately 15% of PCa patients still presented with normal PSA levels or below 4.0 ng/ml [9], which led to false negatives and limited sensitivity for PCa diagnosis because the elevated PSA values were also caused by other non-cancerous factors including age, infection, prostatitis, and benign prostatic hyperplasia (BPH) [10]. Moreover, clinical management of PCa is also highly dependenton the identification of novel biomarkers, which need a more precise prediction of disease progression of PCa. Thus, there is an urgent need to identify sensitive and specific biomarkers for the early detection of PCa, which will accurately discriminate between diagnoses of PCa and BPH in men at a precocious stage for direct early therapeutic/surgical intervention.

Scientists from multiple fields have used different approaches to discover novel differentially expressed genes (DEGs) and miRNAs as potential biomarkers for discriminating PCa from BPH (Supplementary Table 1). Several common altered chromosomal regions, such as deletions on chromosomes 3p14.1-3p13 and 13q13.3-13q14.11, have been identified, and PTP4A3 and ELF1 could be considered possible biomarkers for PCa progression [11]. The expression of Distal-Less Homeobox 1 (DLX1) was found to be much higher in PCa than BPH when used as potential clinical biomarkers for PCa diagnosis, which plays a role in PCa progression [12]. NOS3 was overexpressed in the peripheral blood samples of PCa patients with 5.8-fold higher than BPH through cDNA microarray, which can also be used as a candidate biomarker for PCa progression [13]. To find better serum markers than PSA and potential new therapeutic targets, Stamey determined that 64 down-regulated and 22 up-regulated genes were in Gleason grade 4/5 cancer with HuGeneFL probe arrays [14]. Using human tissue microarrays (TMA), Gomes found that six transmembrane epithelial antigens of the prostate 1 (STEAP1) were highly liable for distinguishing malignant prostate stages from BPH and up-regulated in both plasma membrane and cytoplasm of prostate cancer and PIN lesions, which associated positively with higher Gleason scores [15]. Matos found that thrombospondin 2 (TSP2) was down-regulated in PCa and related to PCa progression, which will be a potential biomarker for PCa from BPH [16]. Leidinger [17] further analyzed the miRNome from blood samples drawn from PCa and BPH patients and found that miR-221-5p and miR-708-3p were down-regulated in PCa compared to BPH. Rane revealed that miR-548c-3p could be a functional biomarker for PCa progression [18]. Specific autoantibody signatures including TARDBP, TLN1, PARK7, LEDGF/PSIP1, and CALD1 and differentially reactive antigens (DIRAGs) also have been reported as biomarkers for distinguishing between PCa and BPH by the native antigen reverse capture microarray platform and the immunome of PCa [19, 20]. However, these biomarkers for a potential application in the diagnosis of PCa did not yield evidence that might substitute or complement PSA. In this study, we analyzed the transcriptomic profiles and functional pathways in FNA biopsies to discriminate PCa from BPH. The purpose of our study was to analyze the genome-wide changes between PCa and BPH and discover novel DEGs for the diagnosis of PCa. Through gene expression profiling analysis and functional pathway analysis, we identified the DEGs as novel potential biomarkers and therapeutic targets for PCa.

## RESULTS

### Gene expression profiling analysis of prostate tumor and BPH tissues

**Figure 1:**
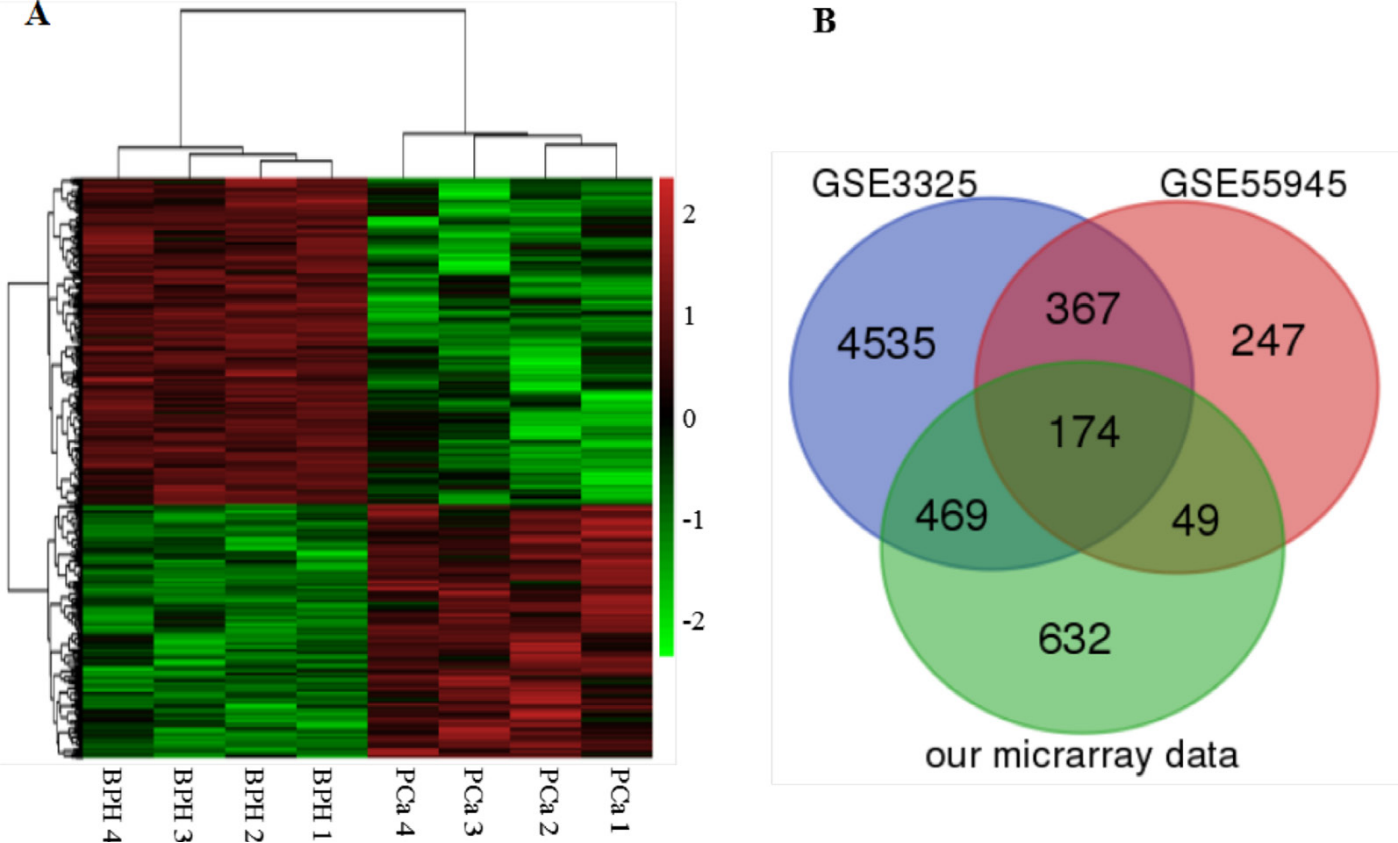
Heat map and Venn diagram showing expression gene profiles (**A**) Heat map. DEGs (FC > 2 and FDR < 0.1) in PCa and BPH tissues are analyzed using hierarchical clustering. Each row represents a single gene and each column represents one sample. Red indicates high relative expression and green indicates low relative expression. (**B**) Venn diagram. Identification of DEGs in PCa using GEO database. The overlapped DEGs in PCa tissues from the expression profiles of GSE3325, GSE55945 and our microarray data.

From prostate needle biopsy specimens, the average RNA amount harvested in FNA biopsies was approximately 2.42 μg (0.66–16.20 μg) for the PCa group and 1.06 μg (0.31–2.31 μg) for the control group derived from BPH tissues. To comprehensively investigate the potential utility of DEGs as PCa biomarkers, first a genome-wide analysis of the gene transcripts expressed in PCa tissues was performed with Affymetrix Human U133 Plus 2 array and revealed that approximately 255 genes (including 132 up-regulated and 123 repressed genes) in prostate tumor tissues exhibited more than a 5.0-fold change in expression level when compared with BPH tissues (*p* ≤ 0.01). Hierarchy cluster analysis also indicated that the 8 samples were distributed into two clusters: 4 PCa samples in one cluster and 4 BPH samples in another cluster (Figure 1A). These results revealed that grouping was reasonable, and the data can be applied directly to further analysis. Next, we analyzed the gene expression profiles of GSE3325 and GSE55945 related to PCa from the gene expression omnibus (GEO) database. We found the dataset GSE3325 included 9 PCa samples, 4 pools of those PCa samples, 4 BPH samples, and 2 pools of the 4 BPH samples. Thus, GSE3325 included 9 PCa tissues and 4 BPH tissues, and GSE55945 included 13 PCa tissues and 8 BPH tissues. All patients’ information was anonymized and de-identified prior to analysis. Finally, we generated fold-change values along with corresponding *p*-values for the gene expression profiles and downloaded gene expression profiling data from the two studies, which included 34 tissue samples (22 PCa samples and 12 BPH tissue samples) excluding samples of the already included benign or prostate samples. These data showed a significant fold-change value in the gene expression of at least one gene. Figure 1B lists the common genes of the two databases from the GEO [21], combined with our microarray results. These 174 common genes are shown by the Venn diagrams, using the false discovery rate (0.05), *p*-values less than 0.05, and two-fold changes, which may be potentially involved in PCa progression from all three microarrays. Although many studies of DEGs as candidate biomarkers of PCa have been published, the reliability of these findings remains uncertain as they were generated from investigations without competent evidence of reproducibility and independent clinical validation.

### qRT-PCR validation of the DEGs in PCa tissue and prostate cancer cells

**Figure 2:**
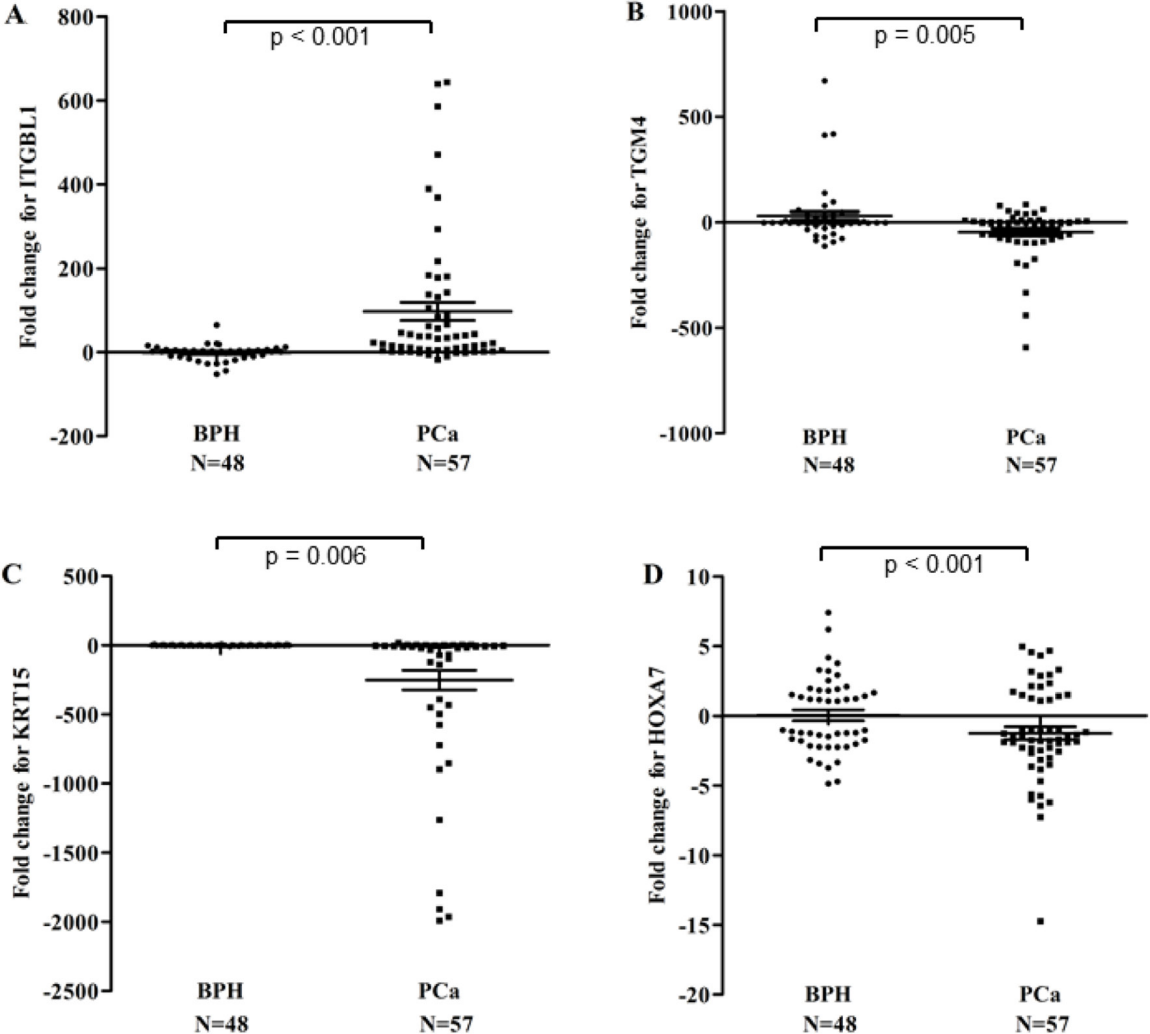
Relative expression scatter plots of the DEGs (ITGBL1 (**A**), TGM4 (**B**), KRT15 (**C**) and HOXA7 (**D**)) in 57 PCa samples compared to 48 BPH tissues. ITGBL1 genes was up-regulated and HOXA7, KRT15 and TGM4 were down-regulated in PCa tissues compared to BPH tissues, confirming the results of the array.

**Figure 3:**
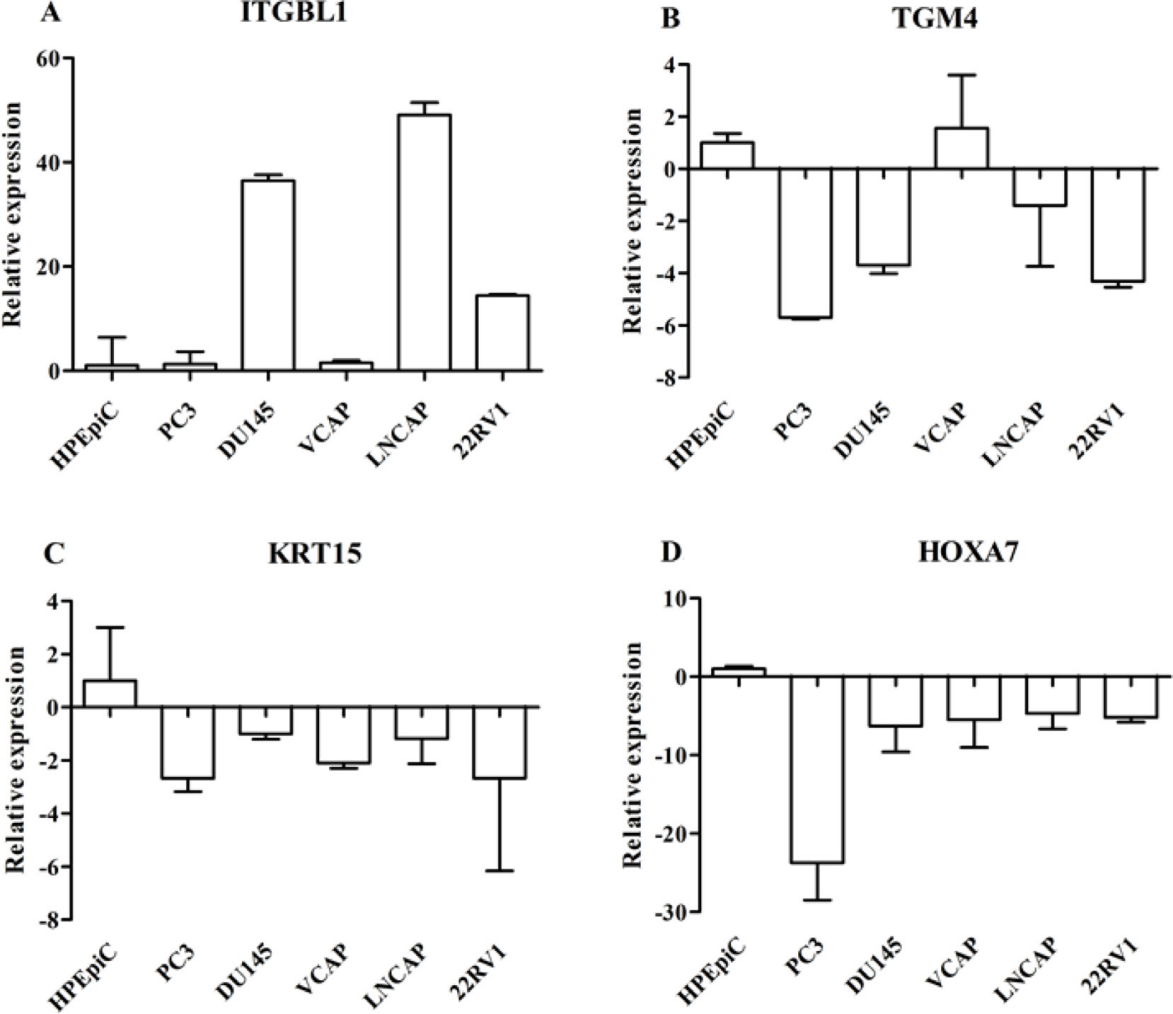
Real-time PCR analysis of DEGs such as ITGBL1 (**A**), TGM4 (**B**), KRT15 (**C**), and HOXA7 (**D**) genes in the prostate cancer cell lines (Vcap, PC3, DU-145, LNcap and 22RV1) and normal human prostate epithelial HPEpiC cells. The expression status of these DEGs was normalized against 18s ribosomal RNA. Data are represented as the mean ± SD of three biological and three technical replicates.

We further validated the DEGs, such as ITGBL1, KRT15, TGM4, and HOXA7 genes, with quantitative real-time polymerase chain reaction (qRT-PCR) in the prostate cell lines (PCa Vcap, PC3, DU-145, LNcap, 22RV1 cells, and the normal human prostate epithelial cells HPEpiC cells) and primary tumors (Figures 2 and 3). qRT-PCR revealed that ITGBL1 was up-regulated and HOXA7, KRT15 and TGM4 were down-regulated in the PCa compared to the BPH tissues (Figure 2). The expression of ITGBL1, HOXA7, KRT15 and TGM4 were significantly different between PCa and BPH tissues (Table 1). We also found that HOXA7 was positively correlated with KRT15 (*r* = 0.328, *p* = 0.001) (Supplementary Table 3). Compared to HPEpiC cells, ITGBL1 was up-regulated in PCa DU145, LNCap and 22RV cells. HOXA7 and KRT15 were repressed in the PCa Vcap, PC3, DU-145, LNcap and 22RV1 cells, and TGM4 was also down-regulated in PC3, DU145, LNcap and 22RV1 cells, which further confirmed the results of our microarray (Figure 3).

**Table 1:** DEGs expression levels in samples of PCa and BPH control

DEGs	PCa	BPH	*P*-Value
ITGBL1 (mean ± SD)	98.6 ± 185.6	−0.6 ± 11.5	0.000
KRT15 (mean ± SD)	−232.2 ± 514.1	0.1 ± 2.6	0.005
HOXA7 (mean ± SD)	−1.3 ± 3.4	0.4 ± 2.7	0.006
TGM4 (mean ± SD)	−50.2 ± 107.7	46.9 ± 136.9	0.000

### Differential expression genes profiles as biomarkers of prostate cancer

**Figure 4:**
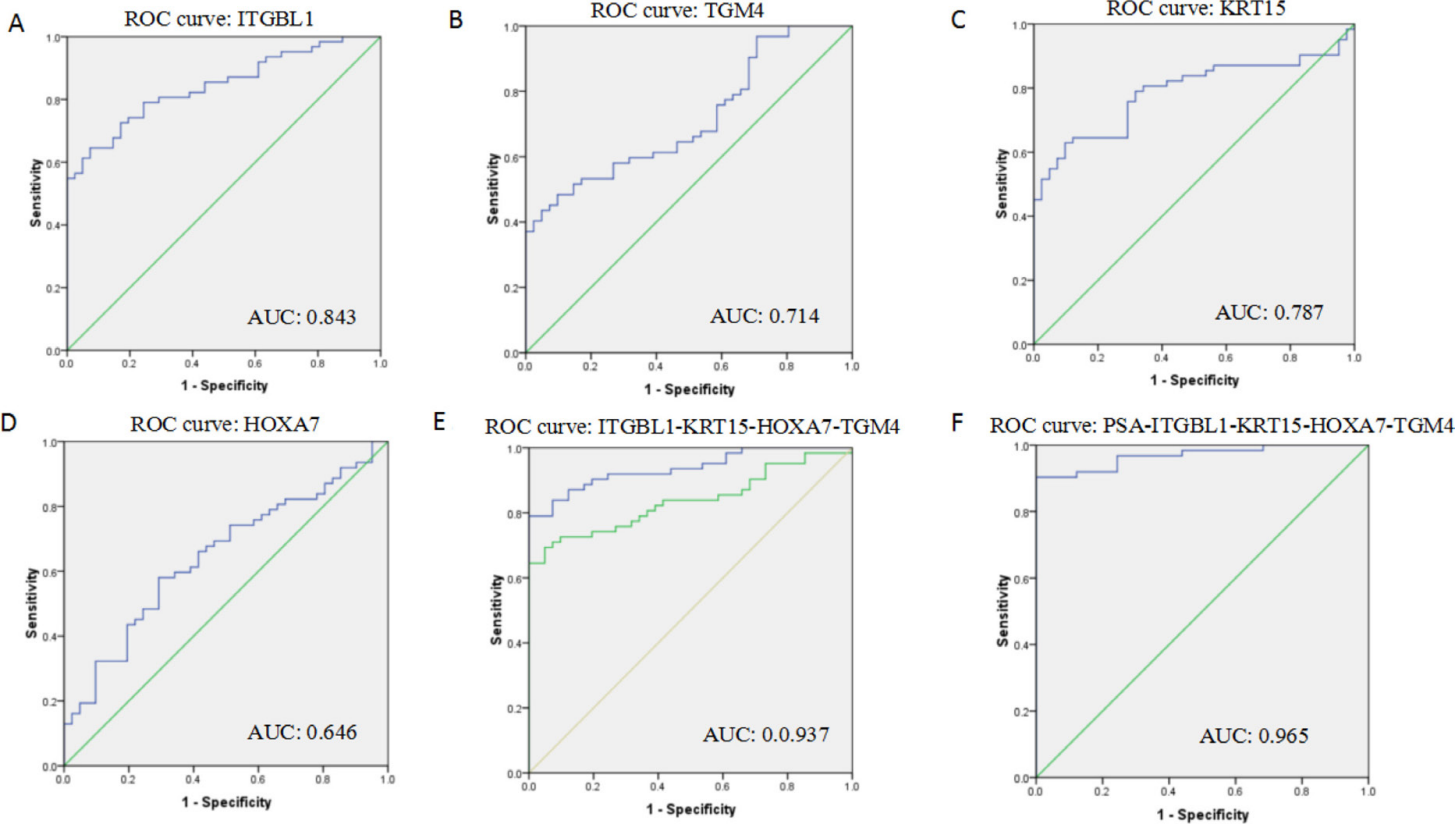
Receiver operating characteristic curves (ROC) showing expression levels of individual DEGs (ITGBL1, KRT15, TGM4, and HOXA7) (**A**–**D**) and the 4 DEGs combination (**E**) in PCa patients and BPH controls; the 4 DEGs and PSA combination (**F**). The curves were compared using univariate (log-rank) analysis.

ROC curves were constructed to determine the ability of the above four DEGs to differentiate PCa samples from BPH samples (Figure 4). The AUC for ITGBL1, TGM4, KRT15 and HOXA7 was 0.843, 0.714, 0.787 and 0.646 (Figure 4A–4D); for all four DEGs combined, the AUC was 0.937 (Figure 4E), documenting that the altered levels of the four DEGs can differentiate patients with PCa from BPH controls. When serum expression of PSA was considered along with the 4 DEGs, the AUC was 0.965 (Figure 4F), which was significantly improved compared to both PSA alone (AUC = 0.822) and the combination of the four DEGs without PSA (AUC = 0.937). ROC curves helped determine the sensitivities and specificities of the DEGs at various cutoff values. Using the optimal cutoff points, sensitivity and specificity were 82.3% and 61.0%, respectively, for ITGBL1; 61.3% and 61.0% for TGM4; 80.6% and 65.9% for KRT15; and 61.3% and 61.0% for HOXA7. The sensitivity and specificity for the 4 DEGs combined were 87.1% and 87.8%, respectively. Finally, the combination of the 4 DEGs plus PSA had a sensitivity of 89.5% and a specificity of 97.6%, which was significantly improved from PSA alone (80.6% sensitivity, 63.4% specificity) for PCa diagnosis (Table 2).

**Table 2:** ROC analysis of the expression levels of individual DEGs (ITGBL1, KRT15, TGM4, and HOXA7) in FNA biopsies and serum PSA in PCa patients and BPH controls

	Sensitivity	Specificity	AUC (95% CI)	*P*-value
Four DEGs + PSA	89.5	97.6	0.965 (0.93–0.998)	0.000
Four DEGs	87.1	87.8	0.937 (0.89–0.98)	0.000
PSA	80.6	63.4	0.822 (0.74–0.91)	0.000
ITGBL1	82.3	61.0	0.843 (0.77–0.92)	0.000
TGM4	61.3	61.0	0.714 (0.19–0.38)	0.000
KRT15	80.6	65.9	0.787 (0.13–0.30)	0.000
HOXA7	61.3	61.0	0.646 (0.25–0.46)	0.013

### Association between the expression of DEGs and clinicopathological factors in PCa patients

Next, the selected DEGs were analyzed in relation to the clinicopathological factors of the PCa patients, and Pearson's correlation coefficient was performed to analyze the correlation between the selected DEGs and glucose and lipid metabolism (Table 3). Our results showed that the increased expression of ITGBL1 correlated with serum total cholesterol (*r* = 0.454, *p* = 0.045) and triglyceride (*r* = 0.500, *P* = 0.025). HOXA7 expression levels were significantly lower in cases with higher fasting plasma glucose (FPG) (*r* = −0.532, *p* = 0.009). TGM4 was inversely related to Gamma-Glutamyltransferase (GGT) (*r* = −0.513, *p* = 0.001). GGT is a membrane-bound enzyme and is involved in biotransformation, nucleic acid metabolism, and tumorigenesis [22]. ITGBL1 was expressed in most patients with PSA > 4 μg/L at remarkably high levels (Supplementary Table 4). Furthermore, there were significant differences in the KRT15 and ITGBL1 expression levels between smoker and non-smoker groups (*p* = 0.025 and *p* = 0.008, respectively) (Supplementary Table 5). We found that KRT15 and TGM4 in alcohol drinkers were expressed at remarkably low levels (*p* = 0.025 and *p* = 0.009, respectively) (Supplementary Table 5).

**Table 3:** Correlation analysis of DEGs, and cholesterol (TC), triglyceride (TG), fasting plasma glucose (FPG) and GGT in the PCa patients

DEGs	TC(mmol/L), *r* (*P*)	TG(mmol/L), *r* (*P*)	FPG(mmol/L), *r* (*P*)	GGT(IU/L), *r* (*P*)
ITGBL1	0.454^*^ (0.045)	0.500^*^ (0.025)	0.109 (0.621)	0.108 (0.531)
KRT15	0.004 (0.988)	−0.144 (0.544)	−0.134 (0.542)	0.134 (0.435)
HOXA7	0.155 (0.514)	−0.336 (0.147)	−0.532^*^ (0.009)	0.108 (0.532)
TGM4	0.089 (0.710)	0.241 (0.306)	0.268 (0.217)	−0.513^*^ (0.001)

Notes: *r*, Pearson correlation; *P*, significance.

### Functional pathway analysis of DEGs in PCa

Finally, we analyzed the biological process of the 255 DEGs of prostate cancer (fold change equal or higher than 5 and *p* < 0.01), which was involved in the cellular process, single-organism process, metabolic process, biological regulation, regulation of cellular process and response to stimulus, and mainly served as protein binding, ion binding, catalytic activity, anion binding and carbohydrate derivative binding from the molecular function analysis. The IPA analysis of DEGs in PCa showed that DEGs mainly participated in DNA damage-induced protein 14-3-3 sigma signaling, mitotic roles of polo-like kinase, GADD45 signaling, hematopoiesis from pluripotent stem cells and is apparent in atherosclerosis signaling (Table 4). A comprehensive network analysis of the DEGs revealed that they were associated with four network functions relevant to the development of cancer, diseases and disorders (Supplementary Figure 1 and Supplementary Table 6), which were associated with the following: the consistency of the cell cycle, cellular assembly and organization; embryonic development, organismal development and developmental disorder; dermatological diseases and conditions, inflammatory disease and inflammatory response; and endocrine system disorders, cardiovascular disease and pulmonary hypertension (Supplementary Figure 1A–1D).

**Table 4:** The top five pathways of DEGs in PCa using IPA analysis

Ingenuity canonical pathways	*P*-Value	Overlap
DNA damage-induced protein 14-3-3 sigma signaling	1.84E-05	21.1% (4/19)
Mitotic roles of polo-like kinase	2.59E-04	7.6% (5/66)
GADD45 signaling	5.46E-04	15.8% (3/19)
Hematopoiesis from pluripotent stem cells	7.04E-04	8.5% (4/47)
Atherosclerosis signaling	7.14E-04	4.8% (6/124)

## DISCUSSION

Prostate cancer is a highly complex and heterogeneous disease that includes genetic aberrations, local invasion of extracellular matrix, and metastasis of prostatic carcinoma [23]. Numerous genes have been analyzed in an attempt to understand the molecular mechanisms involved in the malignant potential of PCa and to identify high-risk populations as well as novel strategies for early detection and prevention in PCa patients. Previous studies had already used PCa and normal prostate epithelial cells for microarray analysis, but could not fully reflect the DEGs changes in PCa patients. Although the ideal method for screening PCa-related DEGs is to perform gene expression profiling analysis using PCa and BPH tissue samples, which are difficult to obtain except by operating on these patients, it was feasible to obtain PCa and BPH tissue samples using FNA biopsies for gene expression analysis without surgery.

In this study, we demonstrated that 255 DEGs were up/down-regulated in prostate tumors compared with BPH tissues; qRT-PCR also confirmed that the DEGs such as ITGBL1, KRT15, TGM4, and HOXA7 genes were up/down-regulated in PCa tissue and cell lines. Moreover, some DEGs associated with the progression of PCa have further been identified in clinical settings as diagnostic biomarkers. TGM4 has been shown to be down-regulated in PCa tissue and almost uniquely expressed in the prostate gland, which could be measured in urinary secretions from PCa patients [24]. HELLS gene known as lymphoid-specific helicase (LSH), which encodes a lymphoid-specific helicase, may be involved with cellular proliferation and may play a role in the development of non-small cell lung carcinoma. Hoogland AM found that the over-expression of HELLS was implicated in the PC progression, which is in line with our study [25]. Tang found that differential expression levels of HOXA7 were correlated with metastasis and prognosis of liver cancer and those levels indicated an acceleration of liver cancer cells migration and invasion [26]. However, the mechanisms underlying the role of HOXA7, inducing PCa to invade and metastasize, remain unclear. Woolf suggested that the KRT15 gene, encoding for CK15, might be a novel marker for urinary tract epithelial precursor cells [27]. ITGBL1 (Integrin, beta-like 1) is a β-integrin-related extracellular matrix protein and contains ten EGF-like repeats that dominate as a gene in the osteoblast-like gene-expression signature [28], which was highly expressed in ovarian cancer tissues and could promote cancer cell migration and invasion [29], and facilitated the acquisition of tumor cell advantages in recruiting, residing, and organ selectivity to the bone during breast cancer metastasis [28]. However, ITGBL1 was down-regulated in non-small cell lung cancer (NSCLC) tissues as a novel tumor suppressor in NSCLC progression [30]. It was interesting that the expression differences of ITGBL1 was statistically significant in BPH and cancerous tissues in our analysis, which was related to total serum cholesterol and triglyceride in patients with PCa. Previous studies have provided evidence supporting a potential role for lipid metabolism in PCa development and found positive associations between total cholesterol and higher grade or more advanced PCa [31, 32]. *In vitro* models, triglyceride-rich remnant like particles can induce carcinogenesis by up-regulating cell signaling pathways such as the MEK/ERK pathway and lipid biosynthesis [33]. Here, we also demonstrated evidence showing that triglycerides might influence the aggressiveness and severity of PCa; further studies are needed to assess the relationship between these lipids and PSA levels in men with PCa, and extensive clinical validation of these novel PCa biomarkers remains one of the most significant challenges.

Life style-related risk factors such as smoking and drinking may influence PCa development and progression. Meta-analysis demonstrated that heavy tobacco use was associated with overall incidence of prostate cancer and more strongly associated with fatal prostate cancer [34]. Increasing alcohol intake was also positively associated with advanced prostate cancer [35]. We also analyzed the association between the expression of DEGs and clinical pathological factors in PCa patients. Our results demonstrated that the expression levels of ITGBL1 were significantly higher in smokers than in non-smokers (*p* = 0.008); KRT15 was significantly down-regulated in non-smokers (*p* = 0.025) (Supplementary Table 5).

In summary, we must emphasize that these novel biomarkers showed significant differential expression between PCa tissue and BPH tissues, however, they may possibly express in other normal and cancerous tissues. Therefore, it cannot be expected that any of these markers can resolve the problems associated with PSA-based early diagnosis of prostate cancer. Our results derived from these assays, especially from genes presumed to be down- or up-regulated, can be quite cryptic and require intensive follow-up studies to confirm the function of candidate genes one by one with traditional experiments.

## MATERIALS AND METHODS

### Cell culture

Human prostatic carcinoma cell lines DU145 (ATCC Number: HTB-81), PC3 (ATCC Number: CRL-1435), VCAP (ATCC Number: CRL-2876), LNCAP (ATCC Number: CRL-1740) and 22RV1 (ATCC Number: CRL-2505) and the human prostatic epithelial cell line (HPEpiC) were purchased from the Culture Collection of the Chinese Academy of Sciences, Shanghai, China (http://www.cellbank.org.cn/). DU145 and PC3 were cultured in MEM (GIBCO, 41500034, Life Technologies) and F-12 (GIBCO, 21700075, Life Technologies), respectively; LNCAP and 22RV1 were maintained in RPMI-1640 (GIBCO, 31800022, Life Technologies); VCAP and HPEpiC were cultured in DMEM (GIBCO, 12800017, Life Technologies); supplemented with 10% fetal bovine serum (FBS) (Invitrogen, GIBCO) at 37°C in 5% CO2.

### Prostate tumor and benign prostatic hyperplasia tissue samples

A total of 105 prostate needle biopsy specimens were obtained from patients who had elevated PSA values or abnormal findings on digital rectal examination with informed consent forms in the Department of Urology of Zhongshan Hospital affiliated to Fudan University from August 2015 to July 2016. The study was approved by the Institutional Ethics Committee for human studies at Zhongshan Hospital, Fudan University. Pathologic diagnosis and Gleason scoring were microscopically reconfirmed by pathologists. No patients recruited into the present study received any treatment prior to surgery. The partial prostate tissues were injected with neutral-buffered formalin (4%) directly after needle biopsy to allow for fast and equal fixation, and processed for routine pathologic diagnosis. All samples were confirmed by haematoxylin-eosin (HE) staining in the Department of Pathology of Zhongshan Hospital. Other parts of the prostate tissues were transported on ice to the RNAsafe Tissue Stabilizer (10604ES60, Yeason) and then stored at −80°C. Of the samples, 57 cases were PCa and 48 were BPH. The corresponding clinical characteristics of patients were summarized in Supplementary Table 2.

### RNA isolation and purification

Total RNA was isolated from PCa cell lines and frozen prostate tumor samples using Trizol reagent (Invitrogen) and RNeasy Mini Kit (Qiagen, USA) following the manufacturer's instructions. RNA quality and quantity was measured using a Nanodrop-1000 (Thermo Scientific, Waltham, MA, USA). RNA integrity by an Agilent Bioanalyzer 2100 (Agilent technologies, Santa Clara, CA, US).

### Gene expression profiles and data analysis

The RNA samples were extracted from four PCa biopsy samplesand four BPH biopsy controls for microarray profiling and performed in Affymetrix Human U133 Plus 2 arrays for gene expression profiling analysis. Microarray data were analyzed using GeneSpring GX 10 (Agilent). The complete microarray datasets have been available on the NCBI Gene Expression Omnibus (GEO Accession Number: GSE104749). Statistical analysis of gene expression microarray data was carried out using the GeneSpring GX software program. Raw data from gene expression files were imported into the program. Post hoc Bonferroni multiple comparison testing was performed to identify statistically significant differences in the expression of genes between PCa samples and BPH RNA samples, with *P* values less than 0.05 considered significant.

### Quantitative real-time PCR (qRT-PCR)

qRT-PCR was performed in five PCa cell lines (DU145, PC3, VCAP, LNCAP and 22RV1), the normal prostate epithelial cell line (HPEpiC), and 105 prostate needle biopsy tissues to assess DEGs expression (see Supplementary Table 7 for primers sequences) using Superscript III reverse transcriptase according to the manufacturer's instructions (Life Technologies) as previously described. qRT-PCRs were performed on a ViiA7 Real-Time PCR System (Applied Biosystems, USA) using SYBR Green Master (Roche) according to the manufacturer's instructions. The internal control was 18 s. Data were normalized using the 18S RNA, and the fold changes of differential expressed genes were calculated using the 2^−ΔΔCt^ method. Data represents the average of three qRT-PCR replicates for each sample from three biological repeats.

### Functional pathway analysis of key differential expression genes (DEGs)

Gene Ontology (GO) and IPA analysis were performed to explore the function of DEGs in PCa. Ingenuity pathway analysis (IPA, QIAGEN Redwood City,www.qiagen.com/ingenuity) was used to assess the gene networks and pathways enrichment of the modulated genes revealed by the microarray analysis. The GO category was classified by Fisher's exact test, and the p-value was corrected by the false discovery rate (FDR) calculation include molecular function (MF), and biological process (BP). Criteria used for the GO and IPA analyses have been described previously [21, 36, 37].

### Statistical analyses

Differences in DEGs expression between the PCa and BPH groups control were compared with Student's *t*-test. Correlation between the expression of DEGs and clinicopathological parameters was measured by the Pearson's correlation coefficient (*r*) to identify the factors that were independent indicators for analysis. The results were regarded as statistically significant at *p* ≤ 0.05. Statistical analysis was performed using the SPSS 20.0 (IBM-SPSS Inc., Chicago, IL, USA), and graphs were built using GraphPad Prism 5.0 software (GraphPad Software Inc., La Jolla, CA, USA). Receiver operating characteristic (ROC) curves were constructed and the area under the curve (AUC) was calculated to evaluate the ability of each individual DEG and PSA either individually or in combination to detect PCa.

## SUPPLEMENTARY TABLES AND FIGURE



## Abbreviations

AUC: area under the curve
BPH: benign prostatic hyperplasia
CTHRC1: Collagen triple helix repeat-containing protein 1
DEGs: Differential expression genes
DTL: Denticleless protein homolog
FNA: Fine-Needle Aspiration
FPG: fasting plasma glucose
GEO: gene expression omnibus
HCC: hepatocellular carcinoma
HOXA7: Homeobox A7
ITGBL1: Integrin, beta-like 1
KRT15: Keratin 15
IPA: Ingenuity pathway analysis
PCa: prostate cancer
PSA: prostate specific antigen
qRT-PCR: quantitative Real-Time polymerase chain reaction
TC: total cholesterol
TG: total triglyceride
TGM4: transglutaminase 4
TSP2: thrombospondin 2
VCAN: versican
