# Supplementary material for: Molecular analyses of prostate tumors for diagnosis of malignancy on fine-needle aspiration biopsies
Source: Oncotarget. 2017 Nov 6;8(62):104761–71. doi: 10.18632/oncotarget.22289 (PMC5739598; doi:10.18632/oncotarget.22289)
Supplement: Supplementary file 1 [file oncotarget-08-104761-s001.pdf]

Molecular analyses of prostate tumors for diagnosis of malignancy on fine-needle aspiration biopsies

SUPPLEMENTARY MATERIALS

Supplementary Table 1: Studies used in microarray analysis between PCa and BPH

Source data	Array	Sample	Significant findings	Reference
Feik *et al.*	Affymetrix GeneChip	Laser capture microdissected cells from PCa and BPH patients	PTP4A3 and ELF1 as possible factors for tumor progression	[9]
Leidinger *et al.*	miRNA microarray	PCa and BPH blood samples	downregulation of hsa-miR-221-5p and hsa-miR-708-3p in PCa compared to BPH	[10]
Matos *et al.*	Human tissue microarray	PCa and BPH tissue samples	TSP2 down-regulated at PCa tissues could be related to PCa progression.	[11]
Gomes *et al.*	Human tissue microarray	PCa and BPH tissue samples	STEAP1 overexpression in PCa and PIN lesions.	[12]
O'Rourke *et al.*	native antigen reverse capture microarray	PCa and BPH tissue samples	The top 5 autoantibody were TARDBP, TLN1, PARK7, LEDGF and CALD1	[13]
Luna-Coronell *et al.*	microarray profiling with IgG	PCa and BPH plasma samples	61 (out of 471) antigens were higher reactive in BPH.	[14]

Supplementary Table 2: Clinicopathological factors in 105 patients with PCa or BPH controls

Characteristics	No. of PCa (*n* = 57)	No. of BPH (*n* = 48)	*p*-value
**Age, *n* (%)**	70.4 ± 7.6	66.3 ± 8.1	**0.011**
≤50	0 (0%)	1 (2.1%)	
51–60	3 (5.2%)	9 (18.8%)	
61–70	25 (43.9%)	22 (45.8%)	
>70	29 (50.9%)	16 (33.3%)	
**PSA, *n* (%)**	261.6 ± 26.3	12.4 ± 7.0	**0.003**
<4.0 ug/l	0 (0%)	1 (2.1%)	
4.0–10 ug/l	6 (10.5%)	17 (35.4%)	
10–20 ug/l	5 (8.7%)	28 (58.3%)	
>20 ug/l	46 (80.7%)	2 (4.2%)	
**Gleason score, *n* (%)**			
Gleason 5~6	10 (17.5%)	-	-
Gleason 7~10	47 (82.5%)		
**pT stage, *n* (%)**			
pT2	41 (71.9%)		
pT3a/b	10 (17.5%)	-	-
pT4	6 (10.5%)		
**lymphovascular invasion, *n* (%)**			
Absent	41 (71.9%)	-	-
Present	16 (28.1%)		
**Metastasis, *n* (%)**			
Absent	18 (31.6%)	-	-
Present	39 (68.4%)		

Supplementary Table 3: Correlation between HOXA7 and KRT15

DEGs	KRT15 (mmol/L), *r* (*P*)
**HOXA7**	0.328 (0.001)

Supplementary Table 4: Correlation analysis of the expression level of ITGBL1 and serum PSA

Genes	PSA < 4.0 ug/l	PSA > 4.0 ug/l	*P*-value
ITGBL1 (mean ± SD)	2.7 ± 1.6	60.2 ± 153.1	0.000

Supplementary Table 5: Effects of tobacco smoke and alcohol on gene expression

Genes	Non-smokers	Smokers	*P*-value
ITGBL1 (mean ± SD)	24.0 ± 22.1	140.0 ± 194.6	0.008
KRT15 (mean ± SD)	–3.6 ± 3.5	–292.9 ± 617.5	0.025

Genes	Non–drinkers	Drinkers	*P*-value
TGM4 (mean ± SD)	11.5 ± 24.2	–74.3 ± 135.0	0.009
KRT15 (mean ± SD)	–4.3 ± 3.1	–292..9 ± 617.5	0.025

Supplementary Table 6: Top four networks of DEGs in PCa using IPA

Top Diseases and Functions	Score	Focus Molecules	Molecules in Network
Cell Cycle, Cellular Assembly and Organization, DNA Replication, Recombination, and Repair	50	26	14-3-3, Akt, APC (complex), BUB1, CAMKK2, CCNB1, CCNE2, CDC6, CDC20, Cdk, CDK1, CDKN3, Cyclin B, DLGAP5, E2f, E2F8, KIF11, KIF20A, Mpf, NCAPG, NDRG2, NUF2, NUSAP1, PBK, PRC1, RRM2, secreted MMP, TFF3, THBS2, TIMP4, TPX2, TRPM4, UBE2C, Vla-4, ZNF750
Embryonic Development, Organismal Development, Developmental Disorder	35	20	Alp, Alpha catenin, AMACR, ASPM, caspase, CCDC68, CD3, CENPF, Creb, CSTA, cytochrome C, DLX1, DST, EZH2, Hdac, Histone h3, Histone h4, HOXD10, HOXD13, Hsp70, ID4, LMNB1, MIR205HG, Notch, PI3K (complex), Rb, SIM2, TCF, TMEM200A, Top2, TOP2A, TOX3, TP63, UHRF1, UPK1A
Dermatological Diseases and Conditions, Inflammatory Disease, Inflammatory Response	30	18	BCR (complex), CD38, Cytokeratin, DMKN, DSC3, EFS, Hat, IFN Beta, Iga, IgG, IgG1, IGHA1, IGHG1, IGHM, IGKC, IGLC1, Igm, IL12 (complex), Immunoglobulin, Interferon alpha, KRT5, KRT13, KRT14, KRT15, MAP2K1/2, Nfat (family), NFkB (complex), Rap1, RNF128, Rsk, SFN, SLC22A4, Tgf beta, TRIM29, UBE2T
Endocrine System Disorders, Cardiovascular Disease, Pulmonary Hypertension	30	18	26s Proteasome, ADCY, Alpha tubulin, BIRC5, CCK, CENPA, Cg, CYP3A5, CYP4F2, DTL, DUOX1, ELAVL2, Histone H1, ID1, Insulin, Jnk, KCNK3, Lh, MEGF10, Mir122a, b, NRXN1, ONECUT2, PDE11A, PDE8B, Pka, Pka catalytic subunit, PLC, PP2A, Proinsulin, PRR11, Rxr, SFRP4, SLC18A2, Smad2/3, TSH

Supplementary Table 7: Primer sequences for differentially expressed genes

Name	Direction	Sequence (5′-3′)	Size of products
DTL-F	+	CCAGCCTTAGTCCAGATGACC	213bp
DTL-R	–	AGCCTCTATTCAAGCGCCAG	
ITGBL1-F	+	TGTGGCAGGTGTAAGTGTGATA	300bp
ITGBL1-R	–	AGTCGGATCAACATCGTGACA	
KRT15-F	+	AGACCTGAGACGCACGATG	252bp
KRT15-R	–	CGGTAAGTAGCGATCTCCTGC	
HOXA7-F	+	CTGAGGCCAATTTCCGCATC	245bp
HOXA7-R	–	CGGACCTTCGTCCTTATGCT	
CTHRC1-F	+	TGGACACCCAACTACAAGCA	214bp
CTHRC1-R	–	CAATGGGAAGAGGTCCTGAACA	

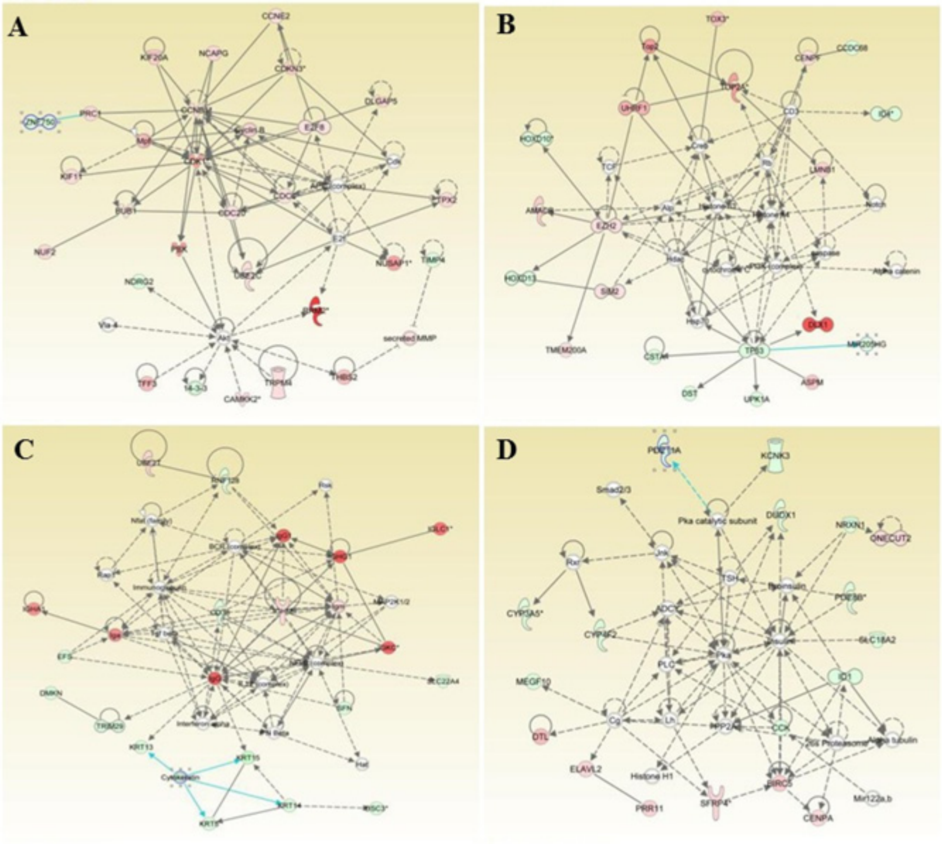

Supplementary Figure 1: Top four significant molecular networks of DEGs in prostate cancer determined by IPA analysis. (**A**) Digestive system development and function, cancer, organismal injury and abnormalities. (**B**) Cell signaling, vitamin and mineral metabolism, nervous system development and function. (**C**) Connective tissue disorders, organismal injury and abnormalities, cancer. (**D**) Cancer, organismal injury and abnormalities, reproductive system disease. Each network was identified based on a numerical rank score according to the degree of relevance of the network to the molecules in the significant genes list and based on the hypergeometric distribution calculated as –log (Fisher's exact test result). In these network illustrations, genes or gene products are represented as nodes, and the biological relationship between two nodes is represented as an edge (line). All edges are supported by at least one published reference.
